# Neurophysiological constraints on the eye-mind link

**DOI:** 10.3389/fnhum.2013.00361

**Published:** 2013-07-15

**Authors:** Erik D. Reichle, Eyal M. Reingold

**Affiliations:** ^1^School of Psychology, University of SouthamptonSouthampton, UK; ^2^Psychology, University of TorontoMississauga, ON, Canada

**Keywords:** ERP, MEG, computational models, reading, saccades

## Abstract

Several current computational models of eye-movement control in reading posit a tight link between the eye and mind, with lexical processing directly triggering most “decisions” about when to start programming a saccade to move the eyes from one word to the next. One potential problem with this theoretical assumption, however, is that it may violate neurophysiological constraints imposed by the time required to encode visual information, complete some amount of lexical processing, and then program a saccade. In this article, we review what has been learned about these timing constraints from studies using ERP and MEG. On the basis of this review, it would appear that the temporal constraints are too severe to permit direct lexical control of eye movements without a significant amount of parafoveal processing (i.e., pre-processing of word *n*+1 from word *n*). This conclusion underscores the degree to which the perceptual, cognitive, and motor processes involved in reading must be highly coordinated to support skilled reading, a par excellence example of a task requiring visual-cognitive expertise.

## Introduction

Reading is one of the most complex tasks that we routinely perform. Part of this complexity reflects the fact that the visual acuity needed to identify the features of printed text seems to be largely limited to a 2° region of the central visual field, the fovea. Because of this limitation, readers must direct their eyes to the majority of words in a text (Rayner, 1998). And although the eyes normally move through the text so rapidly as to make the “task” of moving the eyes appear effortless, this impression is misleading because individual eye movements, called *saccades*, require time to program and execute (Becker and Jürgens, 1979) and are subject to both random and systematic motor error (McConkie et al., 1988). For those reasons, the programming and execution of saccades during reading is itself a highly skilled activity. Adding to this complexity is the fact that the “decisions” about when to move the eyes must be coordinated with the other cognitive processes involved in reading, such as the identification of words and the allocation of covert attention. Attempts to better understand these interactions have produced computational models that describe how lexical processing and attention are coordinated with the programming and execution of saccades to produce the patterns of eye movements that are observed with skilled readers (see Reichle et al., 2003).

Although the assumptions of these models are complex and varied, the two most successful of these models, *E-Z Reader* (Reichle et al., 1998, 2009) and *SWIFT* (Engbert et al., 2002, 2005), posit that the eyes are tightly coupled to the mind, with moment-to-moment “decisions” about when to move the eyes being controlled by lexical processing. For example, according to E-Z Reader, the completion of a preliminary stage of lexical processing (called the *familiarity check*) on a word initiates the programming of a saccade to move the eyes to the next word. And according to the SWIFT model, a saccadic program to move the eyes off of a word is initiated by an autonomous (random) timer that can be inhibited if the fixated word is difficult to process. In this way, both models can explain the ubiquitous finding that difficult (e.g., low frequency) words tend to be the recipients of longer fixations than easy (e.g., high frequency) words (Just and Carpenter, 1980; Inhoff and Rayner, 1986; Rayner and Duffy, 1986; Schilling et al., 1998; Rayner et al., 2004; Kliegl et al., 2006).

Because models of eye-movement control have been used to both simulate the “benchmark” findings related to eye movements in reading and examine various theoretical issues related to reading (e.g., how aging affects readers' eye movements; Laubrock et al., 2006; Rayner et al., 2006), the models represent serious attempts to explain the *eye-mind link*, or interface between lexical processing, on one hand, and eye-movement control, on the other. To the extent that they are successful in this capacity, however, the models raise an important question: How can something as slow as lexical processing mediate the decisions about when to move the eyes? To fully appreciate the paradoxical nature of this question, consider that, although fixation durations are quite variable during reading, occasionally being as short as 50 ms or as long as 800 ms, most are 200–250 ms in duration (Rayner, 1998). Because of this, and because of the fact that some non-trivial amount of time is required to program a saccade (Becker and Jürgens, 1979), it is not immediately obvious how there can be enough time available during each fixation to allow lexical processing to intervene in the decisions about when to move the eyes.

In the remainder of this article, we will attempt to resolve this paradox by reviewing what has been learned from neurophysiology studies about the time course of those processes that are known to play important functional roles in reading and—according to models like E-Z Reader and SWIFT—eye-movement control during reading. The studies that will be reviewed (see Table 1) employ two basic methods, *event-related potentials* (*ERP*s) and *megnetoencephalography* (*MEG*), to examine the times required to propagate visual information from the eyes to the brain, and to then visually encode and engage in lexical processing of printed words. It is important to note, however, that although these studies provide estimates of the times required to complete these processes, these estimates are inherently conservative because they correspond to the first statistically reliable effects of, for example, some variable (e.g., word frequency) on ERP markers of lexical processing. We will therefore provide the minimum, mean, and maximum values of each estimate.

**Table 1:** **Studies (listed chronologically) examining the time course the retina-brain lag, visual encoding, and lexical processing, including their method, task and stimuli, and estimates (in ms) of when the processes occur**.

**Process**	**Study**	**Method**	**Task and Stimuli**	**Mean Estimates [Min, Max] (ms)**
Retina-Brain Lag	Clark et al. (1995)	ERP	viewing checkerboard patterns	42.5 [40–45]
	George et al. (1997)	ERP	recognition of face images	65 [50–80]
	Seeck et al. (1997)	ERP	recognition of face images	70 [50–90]
	Mouchetant-Rostaing et al. (2000)	ERP	recognition of face images	65 [45–85]
	Foxe and Simpson (2002)	ERP	detecting displaced visual disks	57.5 [50–63]
	*Mean Estimates*			*60* [*47–73*]
Visual Encoding	Van Rullen and Thorpe (2001)	ERP	categorizing vehicles vs. animals	77.5 [75–80]
	Foxe and Simpson (2002)	ERP	detecting displaced visual disks	77.5 [70–85]
	Assadollahi and Pulvermüller (2001)	MEG	detecting novel words	105 [90–120]
	Assadollahi and Pulvermüller (2003)	MEG	detecting novel words	90 [60–120]
	Hauk and Pulvermüller (2004)	ERP	lexical decision of letter strings	102.5 [80–125]
	Hauk et al. (2006)	ERP	lexical decision of letter strings	95 [90–100]
	*Mean Estimates*			*91.3* [*77.5–105*]
Lexical Processing	Sereno et al. (1998)	ERP	lexical decision of letter strings	148 [132–164]
	Assadollahi and Pulvermüller (2001)	MEG	detecting novel words	145 [120–170]
	Assadollahi and Pulvermüller (2003)	MEG	detecting novel words	145 [120–170]
	Sereno et al. (2003)	ERP	word-by-word reading	162 [132–192]
	Hauk and Pulvermüller (2004)	ERP	lexical decision of letter strings	170 [150–190]
	Proverbio et al. (2004)	ERP	phoneme detection in words	155 [135–175]
	Baccino and Manunta (2005)	ERP	semantic relatedness judgment	167 [119–215]
	Dambacher et al. (2006)	ERP	natural sentence reading	170 [140–200]
	Hauk et al. (2006)	ERP	lexical decision of letter strings	110 [100–120]
	Penolazzi et al. (2007)	ERP	word-by-word reading	120 [110–130]
	Reichle et al. (2011)	ERP	lexical decision of letter strings	132 [102–162]
	*Mean Estimates*			*147.6* [*126.6–171.8*]

## Results

### Retina-brain lag

The retina-brain lag is the time required for visual information to propagate from the eyes to the earliest cortical areas of the brain. The duration of this lag has been estimated using ERPs by having subjects attend to a visual stimulus (e.g., checkerboard pattern) that is suddenly displayed on a computer monitor and measuring when a *visual-evoked potential* (*VEP*) occurs relative to the onset of the stimulus. Because early cortical areas maintain a retinotopic mapping between spatial locations in the visual field and cortex (Courtney and Ungerleider, 1997), it is possible to localize the neural generators of the VEP and thereby confirm that it reflects early visual processing.

Using this procedure, Clark et al. (1995) found that the VEP had a mean latency of 40–45 ms post-stimulus onset. Using a similar paradigm but having subjects make recognition decisions about images of faces, George et al. (1997) found that VEPs differentiate previously seen versus novel faces as early as 50 ms post-stimulus onset, with these repetition effects peaking at around 80 ms. This finding has been replicated (Mouchetant-Rostaing et al., 2000; Seeck et al., 1997), providing additional evidence that 45–50 ms is sufficient for visual information to reach the brain. Finally, in an experiment designed to examine the time course of both early and late visual processing, Foxe and Simpson (2002) observed a 50–63 ms VEP onset latency when subjects viewed pairs of bilaterally displayed disks with the task of indicating whenever one was displaced from the other. Thus, as Table 1 indicates, estimates of the retina-brain lag range from 47–73 ms across the studies that were reviewed, with a mean of 60 ms.

### Visual encoding

As with the retina-brain lag, the minimal time required to engage in visual encoding has been estimated by examining when differential effects related to the visual properties of stimuli that are suddenly displayed on a computer monitor are first discernable in the ERP record. For example, Van Rullen and Thorpe (2001) had subjects make categorization decisions about photographs of vehicles versus animals and found category-related differences in ERP components as early as 75–80 ms post-stimulus onset. Similarly, Foxe and Simpson (2002) found ERP components that could be localized to the infero-temporal, parietal, and dorsolateral-prefrontal regions (which have been implicated in high-level visual processing; Van Essen and DeYoe, 1995) were active by 70–85 ms post-stimulus onset, suggesting that these higher-level visual-processing regions can modulate visual processing in earlier regions via feedback in as little as 30 ms after processing begins in those earlier regions.

Similar estimates of the time course of visual encoding have also been reported in tasks involving (more global aspects of) lexical processing. For example, Hauk and Pulvermüller (2004) observed effects of word length on ERP components after 80–125 ms when subjects made lexical decisions about short and long letter strings. And using a similar methodology, Hauk et al. (2006) observed word-length effects within 90–100 ms. Two other studies (Assadollahi and Pulvermüller, 2001, 2003) demonstrated the generality of these results by examining the time course of visual encoding of printed words using MEG. In these studies, subjects first memorized a list of short and long high- and low-frequency words and then viewed a random sequence comprised of those words and new words with instructions to press a button whenever they saw a new word. The key finding related to visual encoding were effects of word length, which were evident after 90–120 ms in the first study and after 60–120 ms in the second, indicating that visual properties of the words (i.e., their length) are encoded in as little as 10–40 ms after visual information had been propagated from the eyes to the brain. Thus, as Table 1 shows, the studies reviewed in this section collectively suggest that visual encoding occurs within 77.5–105 ms, with a mean of 91.3 ms.

### Lexical processing

For the purposes of this review, lexical processing will refer to mental operations that convert the visual representation of a word into its (abstract) orthographic form so that that information can be used to access that word's pronunciation and/or meaning. Unfortunately, attempts to determine the time course of lexical processing using neurophysiological methods have produced somewhat inconsistent results. For example, Sereno et al. (1998) conducted a seminal ERP experiment in which subjects made lexical decisions about letter strings that included high- and low-frequency words. (Because the frequency with which a word is encountered in text affects how rapidly its form and meaning can be accessed from memory, word-frequency effects are indicators that lexical processing is well underway; Hudson and Bergman, 1985) Sereno et al. found that word frequency modulated ERP components after only 132–164 ms, suggesting that lexical processing occurs very rapidly. Similarly, Hauk et al. (2006) observed word-frequency effects even earlier, by 100–120 ms.

This conclusion was bolstered by similar findings using both ERP and MEG. For example, the two MEG studies mentioned earlier (in relation to visual encoding) found a word-frequency effect (Assadollahi and Pulvermüller, 2001) and interaction between word frequency and length (Assadollahi and Pulvermüller, 2003) after 120–170 ms. Early lexical effects have also been observed in ERP experiments in which subjects read sentences that were displayed one word at a time: Penolazzi et al. (2007) observed a Frequency × Length interaction after 110–130 ms, and Sereno et al. (2003) observed a frequency effect after 132–192 ms. And similarly, Proverbio et al. (2004) observed word-frequency effects after 135–175 ms in an ERP experiment in which subjects detected target phonemes embedded in visually displayed words.

This evidence for rapid lexical processing must, however, be reconciled with results suggesting that such processing can be much less rapid. For example, Hauk and Pulvermüller (2004) had subjects make lexical decisions about letter strings that contained short and long high- and low-frequency words and found that ERP components were modulated by frequency after 150–190 ms. Similarly, Dambacher et al. (2006) recorded ERPs from subjects who read sentences and found word-frequency effects after 140–200 ms. Consequently, an important challenge for future investigations of the time course of lexical processing would be to isolate the methodological differences that produced the mixed pattern of results in the literature.

Because our goal is to better characterize the relationship between lexical processing and saccadic programming, the final two studies that will be reviewed are particularly important because they were explicitly designed to examine this relationship using ERP. In the first, Baccino and Manunta (2005) had subjects move their eyes to two peripherally-displayed words to make semantic-related judgments about those words. The key finding was that the frequency of the second word modulated ERP components after only 119–215 ms when the data were time-locked to the fixation on the first word, which was interpreted as evidence for rapid parafoveal lexical processing of the second word. In the second study, Reichle et al. (2011) had subjects move their eyes from centrally- to peripherally-displayed letter strings to indicate whether either was a non-word. The key finding was that the frequency of the central word modulated ERP components after only 102–162 ms when the data were time-locked to the onset of the saccade to the peripheral word, which was interpreted as evidence that an early stage of lexical processing initiates saccadic programming. These results, in combination with those of the other studies reviewed in this section, suggest that lexical processing is well under way by 126.6–171.8 ms, with a mean of 147.6 ms. However, it is important to note that, with the exception of Dambacher et al.'s (2006) experiment, these estimates were obtained using non-reading tasks that preclude normal parafoveal processing of upcoming words.

## Discussion

**Figure 1:**
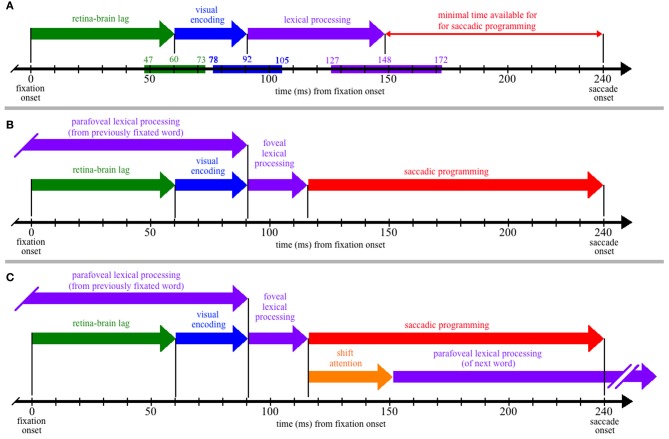
**The time course of processing during a single, 240-ms fixation on a word, including: (1) the propagation of information from the retina to brain (green); (2) visual encoding of the word features (blue); (3) lexical processing (purple); (4) saccadic programming (red); and (5) shifting attention from one word to the next (orange).** (Panel **A**) Neurophysiological estimates for the times required for the retina-brain lag, visual encoding, and lexical processing are indicated by the colored bars superimposed on the time line that is shown at the bottom of the panel, with the three numbers above each colored bar indicating the estimated minimum, mean, and maximal times to complete each respective process (e.g., the estimated minimal time needed for the retina-brain lag is 43 ms). Based on these estimates, there should be little time (92 ms) available for saccadic programming and whatever transmission delays are necessary, e.g., to transmit a signal about the state of lexical processing to the oculomotor system. (Panel **B**) If some amount of lexical processing of the fixated word is actually completed parafoveally, from the previously fixated word, then the amount of (foveal) lexical processing of the word being fixated is reduced (e.g., to 25 ms) and can thereby accommodate more realistic estimates of the time required to program saccades (e.g., approximately 124 ms). (Panel **C**) The time course of processing if one assumes direct lexical control of saccadic programming and the strict serial allocation of attention (e.g., see Reichle, 2011); as shown, the termination of whatever foveal lexical processing is necessary to initiate saccadic programming causes attention to shift to the next word, so that parafoveal lexical processing of that word can begin using visual information acquired from the fixated word. (Note that (Panel **C**) is meant to be theoretically neutral with respect to specific serial-attention models of eye-movement control, and lexical processing is thus shown as a single stage rather than, e.g., being divided into the two stages posited by E-Z Reader. However, the depicted time course maps onto the assumptions of E-Z Reader if: (a) the model's first stage of lexical processing corresponds to whatever lexical processing is completed prior to the initiation of a saccade, and (b) the model's second stage of lexical processing is subsumed in the time required to shift attention.)

To better understand the theoretical implications of this review, it is instructive to superimpose the estimated process durations (Table 1) on a time-line corresponding to the amount of time available for cognitive processing during a single fixation of reading. Figure 1A thus shows the time course of the retina-brain lag, visual encoding, and lexical processing are aligned to the onset of a single 240-ms fixation. (Although this particular duration is arbitrary and ignores the issue of variability, it corresponds to the mean single-fixation duration observed on low-frequency words and is therefore a conservative estimate of the time available for lexical processing during most fixations; Reingold et al., 2012.)

As Figure 1A shows, the neurophysiological estimates suggest that, on average, 148 ms is required to visually encode a printed word and then complete some amount of lexical processing of that word. However, because the fixation is only 240 ms in duration, there is seemingly little time to complete all of the operations that are necessary to move the eyes off of the word 92 ms later. These operations (at a minimum) include the transmission of a signal to the oculomotor system to start programming a saccade, the actual programming of that saccade, and whatever afferent delay occurs in the brainstem circuitry prior to moving the eyes. The conclusion that so little time is available to complete these operations is seemingly at odds with eye-movement experiments suggesting that saccades require 125–200 ms to program (Becker and Jürgens, 1979; Rayner et al., 1983; Reingold et al., 2012). It is also at odds with models of eye-movement control in reading, which posit that lexical processing is the “engine” that cause the eyes to progress through the text (Reichle et al., 1998, 2009; Engbert et al., 2002, 2005). Our analysis of the time course of lexical processing thus poses a paradox if one is to maintain the position that the completion of some amount of lexical processing is what determines when the eyes move during reading.

The solution to this paradox is that a significant portion of the lexical processing of a word that must be completed to “trigger” saccadic programming is actually completed from the preceding word, using visual information that was acquired from the parafovea. How this happens is illustrated in Figure 1B, which is similar to Figure 1A except that lexical processing of the currently fixated word begins from the previously fixated word, so that only 25 ms of lexical processing of the fixated word is actually completed from that word. (Again, this precise value is arbitrary, ignores variability, and is only meant to provide an example.) Under this assumption, there is ample time (~124 ms) for whatever neural transmission is required to signal the oculomotor system to program and then initiate a saccade. This hypothesis about the importance of parafoveal processing is consistent with survival analyses of fixation durations on high- and low-frequency words with versus without parafoveal preview: Word-frequency effects were discernable more than 110 ms earlier with than without preview (Reingold et al., 2012).

Finally, to make this hypothesis more concrete, Figure 1C shows the typical sequence of events that are posited to occur by eye-movement models in which attention is allocated to support the lexical processing of exactly one word at any given time (e.g., E-Z Reader or EMMA; see Reichle, 2011). As shown, the lexical processing of any given word is completed from two locations—from the previously fixated word and from a fixation on the word itself. Then, upon completing whatever lexical processing is necessary to initiate saccadic programming, attention shifts to the next word so that lexical processing of that word can begin using information acquired from the current fixation location. (Note that, according to the E-Z Reader model, the time required to shift attention also includes whatever additional time is needed to complete lexical access of the fixated word.)

Of course, the manner in which E-Z Reader instantiates eye-movement control places the most severe temporal constraints on lexical processing and its coordination with the oculomotor system because only one word is processed at a time. These constraints are significantly relaxed to the extent that multiple words are processed in parallel, as posited by the SWIFT model (Engbert et al., 2002, 2005). According to this alternative theoretical perspective, difficulty associated with processing the fixated word can inhibit the autonomous timer that otherwise initiates a saccadic program to move the eyes to a new viewing location. Recent studies on saccadic inhibition (Reingold and Stampe, 1999, 2000, 2002, 2003, 2004) and prior neurophysiological findings (Munoz et al., 1996) suggest that this type of hypothesized mechanism might produce a very rapid inhibitory effect in as little as 20–30 ms. Consequently, there seems to be ample time for an inhibitory mechanism to intervene in the decisions about when to move the eyes during reading (e.g., see Reingold et al., 2012 for a proposal of a hybrid eye-movement control mechanism incorporating both facilitatory and inhibitory lexical influences).

This review thus indicates that lexical processing is sufficiently rapid to permit direct control of the decisions about when to move the eyes during reading, but that such control also requires a substantial amount of lexical processing from the parafovea—perhaps more that has been acknowledged by reading researchers. This latter conclusion underscores the more basic claim about eye-movement control during reading being a highly skilled activity—one that requires a tremendous degree of coordination between the systems that support attention, word identification, and the programming and execution of saccades.

### Conflict of interest statement

The authors declare that the research was conducted in the absence of any commercial or financial relationships that could be construed as a potential conflict of interest.

## References

[B1] Assadollahi R. Pulvermüller F. (2001). Neuromagnetic evidence for early access to cognitive representations. Neuroreport 12, 207–213 10.1097/00001756-200102120-00007 11209922

[B2] Assadollahi R. Pulvermüller F. (2003). Early influences of word length and frequency: a group study using MEG. Neuroreport 14, 1183–1187 10.1097/00001756-200306110-00016 12821805

[B3] Baccino T. Manunta Y. (2005). Eye-fixation-related potentials: insights into parafoveal processing. J. Psychophysiol. 19, 204–215 10.1027/0269-8803.19.3.204

[B4] Becker W. Jürgens R. (1979). An analysis of the saccadic system by means of double step stimuli. Vis. Res. 19, 967–983 10.1016/0042-6989(79)90222-0 532123

[B5] Clark V. P. Fan S. Hillyard S. A. (1995). Identification of early visual evoked potential generators by retinotopic and topographic analyses. Hum. Brain Mapp. 2, 170–187 10.1002/hbm.460020306

[B6] Courtney S. M. Ungerleider L. G. (1997). What fMRI has taught us about human vision. Curr. Opin. Neurobiol. 7, 554–561 10.1016/S0959-4388(97)80036-0 9287197

[B7] Dambacher M. Kliegl R. Hofmann M. Jacobs A. M. (2006). Frequency and predictability effects on event-related potentials during reading. Brain Res. 1084, 89–103 10.1016/j.brainres.2006.02.010 16545344

[B8] Engbert R. Longtin A. Kliegl R. (2002). A dynamical model of saccade generation in reading based on spatially distributed lexical processing. Vis. Res. 42, 621–636 10.1016/S0042-6989(01)00301-7 11853779

[B9] Engbert R. Nuthmann A. Richter E. Kliegl R. (2005). SWIFT: a dynamical model of saccade generation during reading. Psychol. Rev. 112, 777–813 10.1037/0033-295X.112.4.777 16262468

[B10] Foxe J. J. Simpson G. V. (2002). Flow of activation from V1 to frontal cortex in humans: a framework for defining “early” visual processing. Exp. Brain Res. 142, 139–150 10.1007/s00221-001-0906-7 11797091

[B11] George N. Jemel B. Fiori N. Renault B. (1997). Face and shape repetition effects in humans: a spatio-temporal ERP study. Neuroreport 8, 1417–1423 10.1097/00001756-199704140-00019 9172146

[B12] Hauk O. Davis M. H. Ford M. Pulvermüller F. Marslen-Wilson W. D. (2006). The time course of visual word recognition as revealed by linear analysis of ERP data. Neuroimage 30, 1383–1400 10.1016/j.neuroimage.2005.11.048 16460964

[B13] Hauk O. Pulvermüller F. (2004). Effects of word length and frequency on the human event-related potential. Clin. Neurophysiol. 115, 1090–1103 10.1016/j.clinph.2003.12.020 15066535

[B14] Hudson P. T. Bergman M. W. (1985). Lexical knowledge in word recognition: word length and word frequency in naming and lexical decision tasks. J. Mem. Lang. 24, 46–58 10.1016/0749-596X(85)90015-4

[B15] Inhoff A. W. Rayner K. (1986). Parafoveal word processing during eye fixations in reading: effects of word frequency. Percept. Psychophys. 40, 431–439 10.3758/BF03208203 3808910

[B16] Just M. A. Carpenter P. A. (1980). A theory of reading: om eye fixations to comprehension. Psychol. Rev. 87, 329–354 10.1037/0033-295X.87.4.329 7413885

[B17] Kliegl R. Nuthmann A. Engbert R. (2006). Tracking the mind during reading: the influence of past, present, and future words on fixation durations. J. Exp. Psychol. Gen. 135, 12–35 10.1037/0096-3445.135.1.12 16478314

[B18] Laubrock J. Kliegl R. Engbert R. (2006). SWIFT explorations of age differences in eye movements during reading. Neurosci. Behav. Rev. 30, 872–884 10.1016/j.neubiorev.2006.06.013 16904181

[B19] McConkie G. W. Kerr P. W. Reddix M. D. Zola D. (1988). Eye movement control during reading: I. The location of initial fixations on words. Vis. Res. 28, 1107–1118 10.1016/0042-6989(88)90137-X 3257013

[B20] Mouchetant-Rostaing Y. Giard M.-H. Bentin S. Aguera P.-E. Pernier J. (2000). Neurophysiological correlates of face gender processing in humans. Eur. J. Neurosci. 12, 303–310 10.1046/j.1460-9568.2000.00888.x 10651885

[B21] Munoz D. P. Waitzman D. M. Wurtz R. H. (1996). Activity of neurons in monkey superior colliculus during interrupted saccades. J. Neurophysiol. 75, 2562–2580 8793764 10.1152/jn.1996.75.6.2562

[B22] Penolazzi B. Hauk O. Pulvermüller F. (2007). Early semantic context integration and lexical access as revealed by event-related brain potentials. Biol. Psychol. 74, 374–388 10.1016/j.biopsycho.2006.09.008 17150298

[B23] Proverbio A. M. Vecchi L. Zani A. (2004). From orthography to phonetics: ERP measures of grapheme-to-phoneme conversion mechanisms in reading. J. Cogn. Neurosci. 16, 301–317 10.1162/089892904322984580 15068599

[B24] Rayner K. (1998). Eye movements in reading and information processing: 20 years of research. Psychol. Bull. 124, 372–422 10.1037/0033-2909.124.3.372 9849112

[B25] Rayner K. Ashby J. Pollatsek A. Reichle E. D. (2004). The effects of frequency and predictability on eye fixations in reading: implications for the E-Z Reader model. J. Exp. Psychol. Hum. Percept. Perform. 30, 720–732 10.1037/0096-1523.30.4.720 15301620

[B26] Rayner K. Duffy S. A. (1986). Lexical complexity and fixation times in reading: effects of word frequency, verb complexity, and lexical ambiguity. Mem. Cogn. 14, 191–201 10.3758/BF03197692 3736392

[B28] Rayner K. Reichle E. D. Stroud M. J. Williams C. C. Pollatsek A. (2006). The effects of word frequency, word predictability, and font difficulty on the eye movements of young and older readers. Psychol. Aging 21, 448–465 10.1037/0882-7974.21.3.448 16953709

[B29] Rayner K. Slowiaczek M. L. Clifton C. Bertera J. H. (1983). Latency of sequential eye movements: implications for reading. J. Exp. Psychol. Hum. Percept. Perform. 9, 912–922 10.1037/0096-1523.9.6.912 6227700

[B30] Reichle E. D. (2011). Serial attention models of reading, in Oxford Handbook on Eye Movements, eds Liversedge S. P. Gilchrist I. D. Everling S. (Oxford: Oxford University Press), 767–786

[B31] Reichle E. D. Pollatsek A. Fisher D. L. Rayner K. (1998). Toward a model of eye movement control in reading. Psychol. Rev. 105, 125–157 10.1037/0033-295X.105.1.125 9450374

[B32] Reichle E. D. Rayner K. Pollatsek A. (2003). The E-Z Reader model of eye movement control in reading: comparisons to other models. Behav. Brain Sci. 26, 445–476 10.1017/S0140525X03000104 15067951

[B33] Reichle E. D. Tokowicz N. Liu Y. Perfetti C. A. (2011). Testing and assumption of the E-Z Reader model of eye-movement control in reading: using event-related potentials to examine the familiarity check. Psychophysiology 48, 993–1003 10.1111/j.1469-8986.2011.01169.x 21261631

[B34] Reichle E. D. Warren T. McConnell K. (2009). Using E-Z Reader to model the effects of higher-level language processing on eye movements during reading. Psychon. Bull. Rev. 16, 1–21 10.3758/PBR.16.1.1 19145006 PMC2629133

[B35] Reingold E. M. Reichle E. D. Glaholt M. G. Sheridan H. (2012). Direct lexical control of eye movements in reading: evidence from survival analysis of fixation durations. Cogn. Psychology 65, 177–206 10.1016/j.cogpsych.2012.03.001 22542804 PMC3565237

[B36] Reingold E. M. Stampe D. M. (1999). Saccadic inhibition in complex visual tasks, in Current Oculomotor Research: Physiological and Psychological Aspects, eds Cer W. B. Deubel H. Mergner T. (London: Plenum), 249–255

[B37] Reingold E. M. Stampe D. M. (2000). Saccadic inhibition and gaze contingent research programs, in Reading as a Perceptual Process, eds Kennedy A. Radach R. Heller D. Pynte J. (Amsterdam, Holland: Elsevier), 119–145

[B38] Reingold E. M. Stampe D. M. (2002). Saccadic inhibition in voluntary and reflexive saccades. J. Cogn. Neurosci. 14, 371–388 10.1162/089892902317361903 11970798

[B39] Reingold E. M. Stampe D. M. (2003). Using the saccadic inhibition paradigm to investigate saccadic control in reading, in The mind's Eye: Cognitive and Applied Aspects of Eye Movement Research, eds Hyönä J. Radach R. Deubel H. (Amsterdam, Holland: Elsevier), 347–360

[B40] Reingold E. M. Stampe D. M. (2004). Saccadic inhibition in reading. J. Exp. Psychol. Hum. Percept. Perform. 30, 194–211 10.1037/0096-1523.30.1.194 14769077

[B41] Seeck M. Michel C. M. Mainwaring N. Cosgrove R. Blume H. Ives J. (1997). Evidence for rapid face recognition from human scalp and intracranial electrodes. Neuroreport 8, 2749–2754 10.1097/00001756-199708180-00021 9295112

[B42] Sereno S. C. Brewer C. C. O'Donnell P. J. (2003). Context effects in word recognition: evidence for early interactive processing. Psychol. Sci. 14, 328–333 12807405 10.1111/1467-9280.14471

[B43] Sereno S. C. Rayner K. Posner M. I. (1998). Establishing a time-line of word recognition: evidence from eye movements and event-related potentials. Neuroreport 9, 2195–2200 10.1097/00001756-199807130-00009 9694199

[B44] Schilling H. E. H. Rayner K. Chumbley J. I. (1998). Comparing naming, lexical decision, and eye fixation times: word frequency effects and individual differences. Mem. Cogn. 26, 1270–1281 10.3758/BF03201199 9847550

[B45] Van Essen D. C. DeYoe E. A. (1995). Concurrent processing in the primate visual cortex, in The Cognitive Neurosciences, ed Gazzaniga M. S. (Cambridge, MA: MIT Press), 383–400

[B46] Van Rullen R. Thorpe S. J. (2001). The time course of visual processing: from early perception to decision-making. J. Cogn. Neurosci. 13, 454–461 10.1162/08989290152001880 11388919

